# Physical activity levels across adult life and grip strength in early old age: updating findings from a British birth cohort

**DOI:** 10.1093/ageing/aft124

**Published:** 2013-08-26

**Authors:** Richard Dodds, Diana Kuh, Avan Aihie Sayer, Rachel Cooper

**Affiliations:** 1 Academic Geriatric Medicine, School of Medicine, University of Southampton, Southampton, UK; 2 MRC Lifecourse Epidemiology Unit, University of Southampton, Southampton, UK; 3 MRC Unit for Lifelong Health and Ageing, University College London, London, UK

**Keywords:** physical activity, longitudinal study, grip strength, life course models, older people

## Abstract

**Introduction:** observational studies do not always find positive associations between physical activity and muscle strength despite intervention studies consistently showing that exercise improves strength in older adults. In previous analyses of the MRC National Survey of Health and Development (NSHD), the 1946 British birth cohort, there was no evidence of an association between leisure time physical activity (LTPA) across adulthood and grip strength at age 53. This study tested the hypothesis that cumulative benefits of LTPA across mid-life on grip strength will have emerged by age 60–64.

**Methods:** data from the MRC NSHD were used to investigate the associations between LTPA at ages 36, 43, 53 and 60–64 and grip strength at 60–64. Linear regression models were constructed to examine the effect of activity at each age separately and as a cumulative score, including adjustment for potential confounders and testing of life course hypotheses.

**Results:** there were complete longitudinal data available for 1,645 participants. There was evidence of a cumulative effect of LTPA across mid-life on grip strength at 60–64. Compared with the third of participants who reported the least LTPA participation across the four time points, those in the top third had on average 2.11 kg (95% CI: 0.88, 3.35) stronger grip after adjustments.

**Conclusions:** increased levels of LTPA across mid-life were associated with stronger grip at age 60–64, in both men and women. As these associations have emerged since age 53, it suggests that LTPA across adulthood may prevent decline in grip strength in early old age.

## Introduction

Observational studies do not always find positive associations between physical activity and muscle strength. For example, using data from the Medical Research Council National Survey of Health and Development (MRC NSHD), we found no benefit of increased leisure time physical activity (LTPA) across mid-life on grip strength at age 53 years, despite evidence of cumulative benefits of LTPA on other objective measures of physical capability [1]. This finding was similar to results from other observational studies which have also not found consistent evidence of positive associations between physical activity and strength in both men and women [2–4]. These results are in contrast to the findings from intervention studies showing short-term benefits on strength of resistance exercise training [5], and suggest that the types and intensities of physical activity that people generally undertake in their leisure time may not be sufficient to benefit muscle strength, particularly in the upper limbs, and that the associations may change with age.

The NSHD have since been followed up to age 60–64, a point at which age-related decline in grip strength, typically starting in the fifth decade, would be expected to have begun [6]. The aim of this study was therefore to test the hypothesis that cumulative benefits of increased LTPA in mid-life on grip strength will be evident in the NSHD at age 60–64.

## Methods

### Participants

The MRC NSHD is a socially stratified sample of 5,362 singleton births that took place in 1 week of March 1946 in mainland Britain, with regular follow-up across life [7]. Between 2006 and 2010 (at 60–64 years), 2,856 eligible study members (those known to be alive and with a known address in England, Scotland or Wales) were invited for an assessment at one of six clinical research facilities or to be visited by a research nurse at home. Invitations were not sent to those who had died (*n* = 778), who were living abroad (*n* = 570), had previously withdrawn from the study (*n* = 594) or had been lost to follow-up (*n* = 564). Of those invited, 2,229 (78%) were assessed: 1,690 (59.2%) attended a clinical research facility and the remaining 539 were seen at home [8]. Ethical approval for the study was obtained from the Greater Manchester Local Research Ethics Committee and the Scotland A Research Ethics Committee. Written, informed consent was obtained from study members for each component of the data collection.

### Measurements

Participants were asked about their participation in LTPA at ages 36, 43, 53 and 60–64 years. At age 36, this was based on the Minnesota LTPA questionnaire [9, 10] and at later ages on more basic questions, as described previously [1], with the questions used at 60–64 years the same as those previously described at 53 years [1]. At each age those who reported no participation in LTPA in the previous month or 4 weeks (dependent on the question) were classed as inactive; those participating one to four times as moderately active and those five or more times as most active.

Grip strength was measured in kilograms using an electronic handgrip dynamometer at 60–64 years following a standard protocol [11]. Each participant made three attempts using each hand and the maximum of all six measures was used.

Factors which could act as confounders were identified *a priori*. Educational level at age 26 was categorised into five groups: (i) degree or higher; (ii) A levels, usually attained at age 18 years, or their equivalents; (iii) O levels, usually attained at age 16, or their equivalents; (iv) certificate of secondary education, clerical course, or equivalent and (v) none. Own occupational class at age 53 was used as an indicator of main occupation in adulthood and categorised using the Registrar General's Social Classification into three groups: I or II (high); IIINM or IIIM (medium); IV or V (low). Smoking status at age 53 was categorised into current, never or ex-smoker. Height (cm) and weight (kg) were measured by nurses at age 60–64. Participants were asked at age 60–64 if they had a long-term illness, health problem or disability that limited their ability to carry out everyday activities.

### Statistical analyses

Multiple linear regression models were used to examine the relationships between LTPA at each age and grip strength at 60–64 years, initially adjusted for gender only (model 1). Formal tests of gender interaction were performed in model 1 and where evidence of this was found (*P* < 0.10), subsequent analyses were stratified by gender. A gender-specific *z*-score for grip strength was also produced and tests for gender interaction were repeated using this as the outcome. This did not change the interpretation of the interaction tests. Further adjustments were then made for educational level, occupational class, smoking status, height, weight and presence of limiting disability (model 2). Finally, the LTPA levels at the other three ages were included (model 3). To investigate whether there was evidence of a cumulative effect of LTPA across adulthood, the LTPA variables (in the form 0 = inactive, 1 = moderately active, 2 = most active) were summed across the four time points [to create a score with the range 0 (inactive at all four ages) to 8 (most active at all four ages)]. The lifetime score, modelled as both a continuous score and also as a categorical variable (grouped as 0–1, 2–4 and 5–8) was then used in models 1 and 2.

In a final stage of analyses, we tested whether an accumulation or a sensitive periods model provided the best fit to the data using the structured approach to comparing life course models developed by Mishra *et al.* [1, 12]. This was done by comparing a fully saturated model with accumulation and sensitive periods models (see Supplementary data online, Appendix S1 for details) using the *F*-test statistic.

## Results

One thousand six hundred and forty-five participants had complete data on LTPA at the four ages, grip strength at age 60–64 and covariates. The characteristics of the sample are shown in Table 1. Men reported higher levels of LTPA than women at ages 36 and 43 and had stronger grip at age 60–64. The relationships between physical activity and grip strength are shown in Table 2. In both sexes, being more active at ages 36 and 60–64 was associated with stronger grip, after adjustment for gender (model 1). Being more active at age 53 was also associated with stronger grip but in men only (test of gender interaction, *P* = 0004). These findings attenuated after adjustment for confounders (model 2) but associations with LTPA at 53 (in men) and 60–64 (in both sexes) remained; for example, those who were most active at 60–64 had a mean grip strength 1.73 kg (95% CI: 0.61, 2.85) greater than those who were inactive. There was strong evidence of a linear trend between the cumulative LTPA score and grip strength; a one point increase in the score was associated with a 0.39 kg (95% CI: 0.19, 0.59) stronger grip, after adjustment for confounders (model 2). As shown in Table 2, those in the upper third of cumulative LTPA score (between five and eight) had a mean grip strength 2.11 kg (95% CI: 0.88, 3.35) greater than those in the lower third (score of zero or one), after adjustments. Consistent with this, in comparison with a fully saturated model, an accumulation model where the effect size was allowed to vary between time points best fit the data (*P* = 0.75, see Supplementary data, Appendix S1 for full details).

**Table 1.** Characteristics of the sample, by gender

	Men (*n* = 778)	Women (*n* = 867)	*P*-value*
Physical activity at age [*n* (%)]			
36 years			
Inactive	221 (28.4)	313 (36.1)	0.004
Moderately active	228 (29.3)	227 (26.2)	
Most active	329 (42.3)	327 (37.7)	
43 years			
Inactive	339 (43.6)	444 (51.2)	0.001
Moderately active	190 (24.4)	217 (25.0)	
Most active	249 (32.0)	206 (23.8)	
53 years			
Inactive	327 (42.0)	380 (43.8)	0.27
Moderately active	174 (22.4)	166 (19.2)	
Most active	277 (35.6)	321 (37.0)	
60–64 years			
Inactive	498 (64.0)	537 (61.9)	0.64
Moderately active	108 (13.9)	132 (15.2)	
Most active	172 (22.1)	198 (22.8)	
Grip strength at age: (kg) [mean (SD)]			
53 years (best of four measures)^a^	47.9 (12.2)	28.2 (7.9)	<0.001
60–64 years (best of six measures)^b^	46.2 (11.6)	26.9 (7.4)	<0.001
Covariates			
Height at 60–64 (cm) [mean (SD)]	174.7 (6.5)	161.8 (5.8)	<0.001
Weight at 60–64 (kg) [mean (SD)]	84.9 (13.2)	73.5 (14.9)	<0.001
Limiting disability at 60–64 [*n* (%)]	166 (21.3)	198 (22.8)	0.46
Cigarette smoking at 53 [*n* (%)]			
Current	135 (17.4)	155 (17.9)	0.002
Ex	424 (54.5)	403 (46.5)	
Never	219 (28.2)	309 (35.6)	
Educational level at age 26 [*n* (%)]			
None	234 (30.1)	248 (28.6)	<0.001
CSE, clerical course or equivalent	40 (5.1)	80 (9.2)	
O-level or equivalent	127 (16.3)	232 (26.8)	
A-level or equivalent	240 (30.9)	249 (28.7)	
Degree or higher	137 (17.6)	58 (6.7)	
Occupational class at age 53 [*n* (%)]			
IV or V (low)	73 (9.4)	138 (15.9)	<0.001
III (medium)	264 (33.9)	375 (43.3)	
I or II (high)	441 (56.7)	354 (40.8)	

*From formal test of gender difference using Chi-square test for categorical variables and *t* test for continuous variables.

^a^*n* = 1,590 as 25 men and 30 women in sample did not have a measurement of grip strength at 53 years.

^b^To allow comparison with values from 53 years: mean (SD) grip strength (kg) using best of first four measures at age 60–64: men 44.9 (11.5), women 26.1 (7.3).

CSE, Certificate of Secondary Education.

**Table 2.** Univariable and multivariable regression models for the relationship between physical activity (both at individual time points and as an overall score) and grip strength at age 60–64, including the interaction between physical activity and gender at age 53

Physical activity	Difference in mean grip strength at age 60–64 (kg) (95% CI)					
	Model 1		Model 2		Model 3	
36 years						
Inactive	0		0		0	
Moderately active	1.31	(0.11, 2.51)	1.10	(−0.07, 2.26)	0.82	(−0.36, 2.01)
Most active	1.31	(0.21, 2.41)	1.01	(−0.07, 2.09)	0.38	(−0.78, 1.55)
*P*-value*	0.04		0.10		0.39	
43 years						
Inactive	0		0		0	
Moderately active	0.66	(−0.49, 1.81)	0.26	(−0.86, 1.38)	−0.40	(−1.55, 0.75)
Most active	1.07	(−0.05, 2.18)	0.82	(−0.27, 1.92)	−0.19	(−1.40, 1.02)
*P*-value*	0.15		0.33		0.79	
53 years, stratified by gender (*P*-value** for test of interaction = 0.004)						
Men						
Inactive	0		0		0	
Moderately active	3.76	(2.01, 5.51)	3.01	(1.30, 4.71)	2.69	(0.95, 4.44)
Most active	4.12	(2.60, 5.65)	3.54	(2.05, 5.03)	3.22	(1.66, 4.78)
*P*-value*	<0.001		<0.001		<0.001	
Women						
Inactive	0		0		0	
Moderately active	0.11	(−1.63, 1.84)	−0.20	(−1.90, 1.50)	−0.43	(−2.15, 1.28)
Most active	1.38	(−0.04, 2.79)	0.78	(−0.63, 2.18)	0.38	(−1.09, 1.85)
*P*-value*	0.13		0.42		0.65	
60–64 years						
Inactive	0		0		0	
Moderately active	1.47	(0.13, 2.82)	1.38	(0.07, 2.70)	1.04	(−0.30, 2.39)
Most active	2.18	(1.05, 3.32)	1.73	(0.61, 2.85)	1.18	(−0.02, 2.38)
*P*-value*	<0.001		0.004		0.09	
Lifetime score^a^						
0–1 (*n* = 415)	0		0		*n*/a	
2–4 (*n* = 681)	1.40	(0.23, 2.56)	0.76	(−0.39, 1.91)		
5–8 (*n* = 549)	2.83	(1.61, 4.05)	2.11	(0.88, 3.35)		
*P*-value*	<0.001		0.002			

*n* = 1,645 for all models. Model 1: adjusted for gender only. Model 2: as per model 1 plus height and weight at age 60–64, smoking status at age 53, presence of limiting disability at age 60–64, educational achievement at age 26 and occupational class at age 53. Model 3: as per model 2 plus adjustment for other 3 time points.

^a^Physical activity at each of the four time points summed, where physical activity at each time point is coded as inactive = 0, moderately active = 1 and most active = 2.

*From likelihood ratio test comparing model with physical activity exposure shown to one without.

**From likelihood ratio test comparing model 1 at age 53 with a gender interaction term to one without. The evidence for interaction at age 53 remained when using gender-specific *z*-scores for grip strength (*P* = 0.05). Equivalent tests at all other time points and for the lifetime score had *P-*values >0.10, both using absolute grip strength and gender-specific *z*-scores.

## Discussion

This study has shown evidence of cumulative benefits of increased LTPA across mid-life on grip strength at age 60–64 years. Four other observational studies were identified which had investigated the influence of physical activity on strength at similar ages. In three, there was <5 years between the physical activity and strength assessments [2–4]. The fourth found no association between physical activity at mean age 43 and change in grip strength between mean ages 43 and 64, although becoming sedentary over this period was associated with an accelerated decline in strength [13]. A previous study within the NSHD at age 53 did not find evidence of a cumulative benefit of LTPA [1] and the fact that one has now emerged suggests that LTPA may prevent decline in grip strength in early old age. Additional analyses (Supplementary data online, Appendix S2) support this, with those in the upper third of the cumulative LTPA score to age 53 being less likely than those in the lower third to experience decline in grip strength between 53 and 60–64: odds ratio of decline 0.66 (95% CI: 0.47, 0.92).

Strengths of this study include prospective collection of self-reported LTPA data at multiple ages and the use of an outcome measure that has been shown to predict major ageing outcomes [14–16]. A potential source of bias is the attrition that has occurred in the cohort [8]; however, the earlier null findings [1] at age 53 were unchanged when repeated using the current smaller sample.

This observational study provides additional evidence in support of guidelines which recommend that physical activity should be maintained across adult life [17].

**Key points**

- Intervention studies demonstrate benefits of specific types of exercise for muscle strength in older age.
- Few studies have examined associations between physical activity across mid-life and strength at age 60 or greater.
- This study has shown evidence of a cumulative benefit of greater activity across mid-life on grip strength at age 60–64.
- This suggests that mid-life physical activity may prevent decline in grip strength in early old age.

## Conflicts of interest

None declared.

## Funding

R.D. is supported by a Wellcome Trust Fellowship (Grant number WT099055AIA). R.C. and D.K. are supported by the UK Medical Research Council (Programme code U123092724).

## Supplementary data

Supplementary data mentioned in the text is available to subscribers in *Age and Ageing* online.

Supplementary Data
