# Supplementary material for: Physical activity levels across adult life and grip strength in early old age: updating findings from a British birth cohort
Source: Age Ageing. 2013 Aug 26;42(6):794–8. doi: 10.1093/ageing/aft124 (PMC3809720; doi:10.1093/ageing/aft124)
Supplement: Supplementary Data [file supp_42_6_794__index.html]

Physical activity levels across adult life and grip strength in early old age: updating findings from a British birth cohort — Physical activity levels across adult life and grip strength in early old age: updating findings from a British birth cohort — Supplementary Data 

# Physical activity levels across adult life and grip strength in early old age: updating findings from a British birth cohort

## Supplementary Data

Supplementary Data

**Files in this Data Supplement:**

- Supplementary Data - Docx file
