# Supplementary material for: Physical activity levels across adult life and grip strength in early old age: updating findings from a British birth cohort
Source: Age Ageing. 2013 Aug 26;42(6):794–8. doi: 10.1093/ageing/aft124 (PMC3809720; doi:10.1093/ageing/aft124)
Supplement: Supplementary Data [file supp_aft124_aft124supp.docx]

# Supplementary data

## Appendix 1 – Life course models used

Fully saturated model:

E(Y) = α + β_1_P_1_ + β_2_P_2_ + β_3_P_3_ + β_4_P_4_

+ θ_12_P_1_P_2_ + θ_13_P_1_P_3_ + θ_14_P_1_P_4_ + θ_23_P_2_P_3_ + θ_24_P_2_P_4_ + θ_34_P_3_P_4_ +

+ θ_123_P_1_P_2_P_3_ + θ_124_P_1_P_2_P_4_ + θ_134_P_1_P_3_P_4_ + θ_234_P_2_P_3_P_4_ +

+ θ_1234_P_1_P_2_P_3_P_4_

P_1_..P_4_ represent the leisure-time physical activity responses from each of the four time points (36, 43, 53 and 60-64 years) expressed in binary form (inactive vs moderately/most active).

| Hypothesis and corresponding parameter constraints on regression analysis | Probability from F-test statistic^a^ |
| --- | --- |
| Accumulation – effect size constrained to be the same at each time point  β_1_=β_2_=β_3_=β_4_  and  θ_12_=θ_13_=θ_14_=θ_23_=θ_24_=θ_34_=θ_123_=θ_124_=θ_134_=θ_234_=θ_1234_=0 | 0.50 |
| Accumulation - effect size allowed to vary between time points  β_1_≠β_2_≠β_3_≠β_4_  and  θ_12_=θ_13_=θ_14_=θ_23_=θ_24_=θ_34_=θ_123_=θ_124_=θ_134_=θ_234_=θ_1234_=0 | 0.75 |
| Sensitive period at 36  β_2_=β_3_=β_4_=θ_12_=θ_13_=θ_14_=θ_23_=θ_24_=θ_34_=θ_123_=θ_124_=θ_134_=θ_234_=θ_1234_=0 | 0.05 |
| Sensitive period at 43  β_1_=β_3_=β_4=_θ_12_=θ_13_=θ_14_=θ_23_=θ_24_=θ_34_=θ_123_=θ_124_=θ_134_=θ_234_=θ_1234_=0 | 0.02 |
| Sensitive period at 53  β_1_=β_2_=β_4=_θ_12_=θ_13_=θ_14_=θ_23_=θ_24_=θ_34_=θ_123_=θ_124_=θ_134_=θ_234_=θ_1234_=0 | 0.42 |
| Sensitive period at 60-64  β_1_=β_2_=β_3=_θ_12_=θ_13_=θ_14_=θ_23_=θ_24_=θ_34_=θ_123_=θ_124_=θ_134_=θ_234_=θ_1234_=0 | 0.21 |

All life course models included potential confounders (gender, height and weight at age 60-64, smoking status at age 53, presence of limiting disability at age 60-64, educational achievement at age 26 and occupational class at age 53).

^a^Higher *P*-values indicate that the life course model shown fits the data as well as the fully saturated model.

# *Appendix 2 – Analyses of change in grip strength*

**Changes in grip strength between 53 and 60-64 years, by gender**

|  | **Men**  **(n=753)** | **Women**  **(n=832)** | ***p*-value^a^** |
| --- | --- | --- | --- |
|  |  |  |  |
| **Grip strength [kg] (Mean (SD))** |  |  |  |
| Age 53: best of all 4 measures | 47.9 (12.2) | 28.2 (7.9) | < 0.001 |
| Age 60-64: best of first 4 measures | 45.0 (11.5) | 26.1 (7.3) | < 0.001 |
|  |  |  |  |
| **Relative change in grip strength between 53 and 60-64 (%)^b^** |  |  |  |
| Median (IQR) change | 6.1 (-11.4, 20.1) | 8.4 (-11.7, 24.1) |  |
| Cut-point for top 15^th^ centile with greatest relative change | 28.6 | 35.0 |  |

^a^From formal test of gender difference using *t* test.

^b^Calculated as ((grip at age 53 – grip at age 60-64) / grip at age 53) x 100, such that higher positive values represent greater relative decline between ages 53 and 60-64.

**Logistic regression models for the relationship between lifetime physical activity score and odds of experiencing greatest (top 15th centile) gender-specific relative decline in grip strength between ages 53 and 60-64**

|  | **Odds ratio of experiencing greatest (top 15^th^ centile) gender-specific relative decline in grip strength** | | | |
| --- | --- | --- | --- | --- |
| **Lifetime physical activity score^a^** | Model 1 | | Model 2 | |
|  |  |  |  |  |
| **Per unit increase** | 0.92 | [0.85,0.99] | 0.95 | [0.88,1.02] |
| *P*-value^b^ | *0.02* |  | *0.16* |  |
|  |  |  |  |  |
| **By category** |  |  |  |  |
| 0-1 (n=453) | 1 |  | 1 |  |
| 2-3 (n=520) | 0.66 | [0.47,0.94] | 0.70 | [0.49,0.99] |
| 4-6 (n=612) | 0.66 | [0.47,0.92] | 0.75 | [0.53,1.06] |
| *P*-value^b^ | *0.02* |  | *0.11* |  |

n=1,585 for all models. Model 1: Adjusted for gender only. Model 2: As per model 1 plus height and weight at age 53, smoking status at age 53, presence of life threatening illness at age 53, educational achievement at age 26 and occupational class at age 53.

^a^Physical activity at each of the three time points (36, 43 and 53 years) summed, where physical activity at each time point is coded as inactive = 0, moderately active = 1 and most active = 2.

^b^From likelihood ratio test comparing model with physical activity exposure shown to one without.
